# Correlational analysis of bone health status and vitamin D-related biomarkers in women working in agriculture

**DOI:** 10.1097/MD.0000000000027071

**Published:** 2021-08-27

**Authors:** Min-Chul Cho, Ki Soo Park, Jeong Kyu Shin, Soon Ae Lee, In Ae Cho, Hyen Chul Jo, Seung Chan Kim, Won Jun Choi

**Affiliations:** a Department of Laboratory Medicine, Gyeongsang National University College of Medicine and Gyeongsang National University Hospital, Republic of Korea; b Institute of Health Sciences, Gyeongsang National University, Jinju, Republic of Korea; c Department of Preventive Medicine, Gyeongsang National University College of Medicine, Republic of Korea; d Department of Obstetrics and Gynecology, Gyeongsang National University College of Medicine and Gyeongsang National University Hospital, Republic of Korea; e Department of Obstetrics and Gynecology, Gyeongsang National University Hospital, Republic of Korea; f Department of Obstetrics and Gynecology, Gyeongsang National University Changwon Hospital, Republic of Korea; g Biostatistics Cooperation Center, Gyeongsang National University Hospital, Jinju, Republic of Korea.

**Keywords:** 25-hydroxy vitamin D, agriculture, bioavailable vitamin D, bone health, bone mineral density, vitamin D, vitamin D-binding protein

## Abstract

The purpose of this study was to investigate the status of bone health in women working in agriculture and analyze the associations between bone health and various vitamin D-related biomarkers.

This observational study enrolled women working in agriculture (n = 210) and control occupations (n = 180). The concentration of serum total 25-hydroxy vitamin D [25(OH)D] was measured using the Elecsys Vitamin D Total Kit, and serum vitamin D-binding protein (VDBP) was measured by enzyme-linked immunosorbent assay. Along with albumin, 25(OH)D and VDBP were used to calculate the concentrations of bioavailable and free 25(OH)D. Bone mineral density (BMD) and T-score were measured at lumbar 1 to 4 and the femur neck using dual-energy X-ray absorptiometry. To identify factors affecting BMD, log-linear model and linear regression analysis were performed for statistical analysis.

Agricultural women workers showed higher serum concentrations of bioavailable 25(OH)D (12.8 ± 3.7 vs 8.7 ± 5.1 ng/mL) and lower VDBP concentrations (201.8 ± 45.0 vs 216.0 ± 68.2 μg/mL) than control women. The association between these 2 vitamin D related-biomarkers and femur neck BMD were confirmed through univariable and multivariable linear model analysis. Although lumbar BMD did not differ between groups, the agricultural group displayed a lower femur BMD and a 4.3-fold increase in the risk of osteoporosis compared with the control group.

Women working in agriculture showed lower femur BMD than the control group. Of the vitamin D-related biomarkers tested, bioavailable 25(OH)D and VDBP were associated with BMD. As bioavailable 25(OH)D levels are affected mainly by VDBP levels, VDBP may play a role in the lower femur neck BMD values observed in the agricultural group. Thus, the measurement of VDBP concentration might be considered a simple and non-invasive method for measuring bone health status.

## 1 Introduction

A variety of factors affect bone health, including genetics, nutrition, physical activity, hormones, and vitamin D levels.^[1–4]^ Vitamin D deficiency impairs not only bone mineralization and calcium absorption but also muscle strength and balance, which may increase the risk of falling.^[5]^ Research on the connection between bone health and vitamin D demonstrates the importance of adequate vitamin D levels in the prevention of fractures in patients with osteoporosis.^[6]^ Additionally, osteoporosis risk decreases with increased serum levels of 25-hydroxy vitamin D [25(OH)D], a vitamin D metabolite.^[7]^

Vitamin D is either produced in the skin or ingested from dietary sources and plays an essential role in bone metabolism, as well as other biological processes. The hydroxylation of vitamin D to 25(OH)D occurs in the liver, catalyzed by 1 of several cytochrome P450 enzymes.^[8]^ The 1α-hydroxylase enzyme, expressed predominantly in the kidneys, catalyzes the conversion of circulating 25(OH)D to an active form of vitamin D, 1,25-dihydroxy vitamin D, which regulates numerous genes responsible for cellular proliferation, differentiation, apoptosis, and angiogenesis.^[9]^ Patients with vitamin D deficiency are at increased risk for diseases, including osteoporosis, musculoskeletal pain, and spine pain. Serum 25(OH)D declines with age, and vitamin D deficiency may contribute to bone loss in women, especially after menopause.^[10]^ Typically, vitamin D deficiency is found in countries with insufficient sun exposure during the winter months.

Traditionally, the vitamin D status of an individual has been assessed by measuring the total serum concentration of 25(OH)D, with vitamin D deficiency defined as less than 20 ng/mL and vitamin D insufficiency defined as 20 to 30 ng/mL.^[11–13]^ Recent studies have questioned the accuracy of using 25(OH)D alone to define vitamin D status, although studies correlating 25(OH)D with bone density and fractures have reported mixed findings.^[14–17]^ Several recent studies have sought to identify other indicators that accurately reflect vitamin D status, and the level of bioavailable 25(OH)D has emerged as a promising candidate.

The free hormone hypothesis postulates that only hormones free from binding proteins are able to enter cells and induce biological activity.^[18]^ The majority of 25(OH)D and 1,25-dihydroxy vitamin D circulate bound to vitamin D-binding protein (VDBP), a vitamin D transporter best known for its role in the circulation of vitamin D metabolites. VDBP is a glycosylated alpha-2-globulin protein that is encoded by the *GC* gene and is produced by the liver.^[19]^ Circulating 25(OH)D or 1,25-dihydroxy vitamin D can exist in 3 forms: bound to VDBP (85%–90%), bound to albumin (10%–15%), or in a free unbound form (<1%).^[20,21]^ Bioavailable 25(OH)D is defined as the fraction of 25(OH)D that is not bound to VDBP, including both free and albumin-bound forms. The significance of bioavailable 25(OH)D has been suggested by studies reporting stronger correlations for various parameters, such as serum calcium concentration, parathyroid hormone level, bone mineral density (BMD), and vascular outcomes, with bioavailable 25(OH)D than with total 25(OH)D.^[22–24]^

A limited number of studies have been conducted examining the impacts of jobs on bone health; however, correlations between occupational activity and BMD exist.^[25,26]^ Although women working in agriculture are exposed to hard physical work, which can overload the joints, no study has compared their bone health status with that of women working in other occupations. In addition, few studies have examined the correlation between bone health and vitamin D-related biomarkers, such as VDBP and 25(OH)D, in bioavailable and free forms. In the present study, we investigated bone health and vitamin D-related biomarkers in women employed in agricultural occupations.

## 2 Material and methods

### 2.1 Study subjects

This study included 210 healthy women working in agriculture and 180 healthy women with other occupations in the city of Jinju. All participants working in agriculture were registered with the National Farmers Health Checkup Program, a free government-funded program. The professions of the 180 occupational controls were as follows: 55 homemakers, 61 clerks, 41 administrators, 22 self-employed, and 1 unemployed. All participants visited the Total Healthcare Service Center of Gyeongsang National University Hospital for a health checkup between May and September 2019. This study has undergone an Ethics Review and was approved by the Gyeongsang National University Hospital Institutional Review Board (GIRB-A16-Y-0012).

### 2.2 Demographic and medical data collection

The study was performed retrospectively through the analysis of medical records. Menopause was defined as the cessation of menstruation for more than 1 year. Women diagnosed with osteoporosis or receiving medications, such as calcium or bisphosphonate, were excluded. In addition, women who have had hyperthyroidism, hyperparathyroidism, rheumatoid joint disease, cancer, kidney disease, and long-term steroid use, as well as women with artificial implants inserted into the spine due to spinal disease, were also excluded.

During the health checkup at the Total Healthcare Service Center, self-reported questionnaires were administered to obtain medical history and lifestyle information, including history of disease, medication history, smoking status, alcohol consumption, and physical activity. Smoking status was defined as more than 1 cigarette per day, and alcohol consumption was defined as drinking more than once a month. Height and weight were measured while standing barefoot with the outer garment off using an automatic height-weight-body mass index (BMI)-measuring device (JENIX DS-102; DS medical JENIX, Korea). The BMI was calculated by dividing body weight (kg) by the square of the height (m^2^). All body measurements were expressed to 1 decimal place.

### 2.3 Diagnosis of osteopenia and osteoporosis

BMD was measured with dual-energy X-ray absorptiometry (Horizon CI, HOLOGIC, MA) in the lumbar spine (vertebrae L1–L4) and femur neck and read according to the International Society for Clinical Densitometry guidelines. The accuracy of the instrument was measured twice in 30 participants. The precision error at the spine was 0.007 g/cm^2^, and the least significant change was measured as 0.019 g/cm^2^ at the 95% confidence interval. BMD was classified as follows: normal (T-score ≥ −1.0 SD), osteopenia (T-score −1.0 to −2.5 SD), and osteoporosis (T-score ≤ 2.5).

### 2.4 Vitamin D and VDBP measurements

Each serum sample was aliquoted into 2 tubes and stored at −80°C until analysis of total 25(OH)D and VDBP. Serum total 25(OH)D concentrations were analyzed using the Elecsys Vitamin D Total Kit with the Cobas e602 module (Roche Diagnostics, Mannheim, Germany); this electrochemiluminescent assay is based on ruthenium-labeled VDBP, biotin-labeled vitamin D, and streptavidin-coated microparticles. VDBP concentrations were measured using the Human Vitamin D BP Quantikine enzyme-linked immunosorbent assay kit (R&D Systems, Minneapolis, MN), according to the manufacturer's protocol.

### 2.5 Calculation of bioavailable and free 25(OH)D

Bioavailable and free 25(OH)D concentrations were calculated using equations reported in previous studies and based on a total 25(OH)D, VDBP, and albumin concentrations derived from the medical records.^[27,28]^

### 2.6 Statistical analysis

All analyses were performed with R version ‘4.0.3’ and were analyzed using a significance level of *P* = .05. For the data presented in Tables 1 and 2, the following analyses were used to compare differences between groups according to the distribution: quantitative data were analyzed using analysis of variance, *t* test, the Kruskal–Wallis test, and the Mann–Whitney *U* test (Wilcoxon rank-sum test); qualitative data were analyzed using the Chi-square test and Fisher exact test.

**Table 1:** Characteristics of patients enrolled in the study.

Variables	Control (n = 180)	Agriculture (n = 210)	Total (n = 390)	*P* value
Age	54.2 ± 7.7	54.7 ± 9.2	54.5 ± 8.5	.929
Height (cm)	159.0 ± 5.1	155.7 ± 5.9	157.2 ± 5.8	.000
Body weight (kg)	59.6 ± 8.8	60.0 ± 9.6	59.8 ± 9.2	.818
BMI (kg/m^2^)	23.6 ± 3.3	24.7 ± 3.8	24.2 ± 3.6	.002
Parity	2.7 ± 1.1	2.8 ± 1.3	2.7 ± 2.2	.152
Smoking				.541
Smoking = “No”	166 (92.2%)	198 (94.3%)	364 (93.3%)	
Smoking = “Yes”	14 (7.8%)	12 (5.7%)	26 (6.7%)	
Alcohol (>1/month)				.349
Alcohol = “No”	84 (46.7%)	87 (41.4%)	171 (43.8%)	
Alcohol = “Yes”	96 (53.3%)	123 (58.6%)	219 (56.3%)	
Menopause				.818
Menopause = “No”	52 (28.9%)	64 (30.5)	116 9 (29.7%)	
Menopause = “Yes”	128 (71.1%)	143 (69.5%)	274 (70.3%)	
Duration of menopause	5.4 ± 5.1	6.6 ± 7.7	6.0 ± 6.6	.702

Values are presented as the mean ± standard deviation. BMI = body mass index.

**Table 2:** Laboratory findings, vitamin D biomarkers, and BMD of patients enrolled in the study.

Variables	Control (n = 180)	Agriculture (n = 210)	Total (n = 390)	*P* value
Calcium (mg/dL)	9.4 ± 0.4	9.3 ± 0.3	9.4 ± 0.4	.103
Phosphate (mg/dL)	3.6 ± 0.5	3.7 ± 0.5	3.6 ± 0.5	.154
Albumin (g/dL)	4.6 ± 0.2	4.4 ± 0.2	4.4 ± 0.3	.000
Total 25(OH)D (ng/mL)	23.5 ± 9.6	22.2 ± 9.9	22.8 ± 9.8	.132
Bioavailable 25(OH)D (ng/mL)	8.7 ± 5.1	12.8 ± 3.7	10.9 ± 4.8	.000
Free 25(OH)D (pg/mL)	21.2 ± 12.5	32.4 ± 9.5	27.2 ± 12.3	.000
VDBP (μg/mL)	216.0 ± 68.2	201.8 ± 45.0	208.3 ± 57.3	.013
BMD (g/cm^2^)				
L1–L4	1.1 ± 0.2	1.1 ± 0.3	1.1 ± 0.2	.252
Femur neck	1.0 ± 0.2	0.9 ± 0.2	0.9 ± 0.2	.012

Values are presented as the mean ± standard deviation. 25(OH)D = 25-hydroxy vitamin D, BMD = bone mineral density, L1–L4 = lumbar vertebrae 1 to 4, VDBP = vitamin D-binding protein.

For the data presented in Table 3, a log-linear model was constructed as a model for the frequency of the contingency table to reveal the relationship between variables. To fit the optimal model, a variable selection process was performed using the backward elimination method. The form of the optimal model is as follows:

**Figure:**
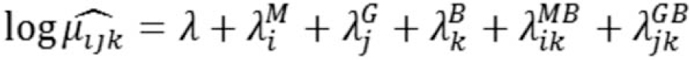



(M=Menopause,G=Group,B=Bone loss;i=[16],j=[16],k=[17])

**Table 3:** Proportions and result of log-linear model for osteopenia and osteoporosis between control and agricultural groups.

	Group	Normal (n = 247)	Osteopenia (n = 104)	Osteoporosis (n = 39)
Menopause “No”	Control	48 (92.3%)	4 (7.7%)	0 (0.0%)
	Agriculture	58 (90.6%)	6 (9.4%)	0 (0.0%)
Menopause “Yes”	Control	72 (56.2%)	49 (38.3%)	7 (5.5%)
	Agriculture	69 (47.3%)	45 (30.8%)	32 (21.9%)

Variables	OR (95% confidence interval)	*P* value
Menopause∗Osteopenia	7.1 (3.7, 15.1)	.000
Agriculture∗Osteopenia	0.9 (0.6, 1.4)	.684
Agriculture∗Osteoporosis	4.3 (1.9, 11.0)	.001

OR = odds ratio.

For the data presented in Table 4, L1–L4 BMD (g/cm^2^) and femur neck BMD (g/cm^2^) were considered as the outcomes, and univariable linear regression analysis was performed. For the data presented in Table 5, to fit the optimal model, L1–L4 BMD (g/cm^2^) and femur neck BMD (g/cm^2^) were considered as the outcomes, and LASSO and stepAIC were used for model building.

**Table 4:** Coefficients of the univariable linear model for the general characteristics and vitamin D variables that affect the BMD of the femur neck and lumbar spine (L1–L4).

Location	Variable	Beta	*P* value	Adjusted R^2^
Femur neck	Age	−0.01	.000	0.171
	BMI	0.003	.250	0.001
	Smoking	−0.136	.001	0.026
	Alcohol	−0.01	.642	−0.002
	Agriculture	−0.071	.000	0.028
	Menopause	−0.166	.000	0.139
	Bioavailable 25(OH)D	0.005	.011	0.014
	Free 25(OH)D	0.002	.034	0.009
	Total 25(OH)D	−0.002	.136	0.003
	VDBP	0	.029	0.010
	Calcium	−0.071	.013	0.013
	Phosphate	−0.043	.049	0.007
	Albumin	0.057	.166	0.002
Lumbar spine	Age	−0.012	.000	0.221
	BMI	0.007	.031	0.009
	Smoking	−0.166	.000	0.031
	Alcohol	0.002	.936	−0.003
	Agriculture	0.006	.790	−0.002
	Menopause	−0.204	.000	0.169
	Bioavailable 25(OH)D	0.007	.001	0.023
	Free 25(OH)D	0.003	.004	0.019
	Total 25(OH)D	−0.001	.212	0.001
	VDBP	0	.035	0.010
	Calcium	−0.074	.020	0.011
	Phosphate	−0.054	.027	0.010
	Albumin	−0.016	.730	−0.002

25(OH)D = 25-hydroxy vitamin D, BMD = bone mineral density, BMI = body mass index, L1–L4 = lumbar vertebrae 1 to 4, VDBP = vitamin D-binding protein.

**Table 5:** Coefficients of the multivariable linear model for characteristics and vitamin D biomarkers affecting the BMD of the femur neck and lumbar spine (L1–L4).

Location	Variable	Beta	*P* value	Adjusted R^2^
Femur neck	Agriculture	−0.117	.000	0.254
	Menopause	−0.078	.006	
	Bioavailable 25(OH)D	0.008	.000	
	Free 25(OH) D	–	–	
	Total 25(OH) D	–	–	
	VDBP	0.001	.000	
Lumbar spine	Agriculture	−0.036	.124	0.263
	Menopause	−0.069	.027	
	Bioavailable 25(OH)D	0.006	.009	
	Free 25(OH)D	–	–	
	Total 25(OH)D	–	–	
	VDBP	0.002	.000	

25(OH)D = 25-hydroxy vitamin D, BMD = bone mineral density, L1–L4 = lumbar vertebrae 1 to 4, VDBP = vitamin D-binding protein.

## 3 Results

### 3.1 Demographic and clinical characteristics

The BMI was higher in the agriculture group (24.7 ± 3.8 kg/m^2^) than in the control group (23.6 ± 3.3 kg/m^2^; *P* = .002), although only the heights between groups differed (*P* = .000), with no difference in weights (Table 1). No significant differences in parity, drinking and smoking history, or the presence of menopause were identified between the 2 groups (Table 1).

### 3.2 Laboratory findings, vitamin D biomarkers, and BMD

Serum concentrations of calcium and phosphorus did not differ between the 2 groups; however, the albumin concentration was lower in the agriculture group (4.4 ± 0.2 g/dL) than in the control group (4.6 ± 0.2 g/dL; *P* = .000; Table 2). Albumin concentrations in both groups were within the normal reference range (3.5–5.2 g/dL).

BMD did not differ between groups in L1–L4; however, a significantly lower femur neck BMD was observed for the agriculture group (0.9 ± 0.2 g/cm^2^) compared with that in the control group (1.0 ± 0.2 g/cm^2^; *P* = .012; Table 2).

**Figure 1:**
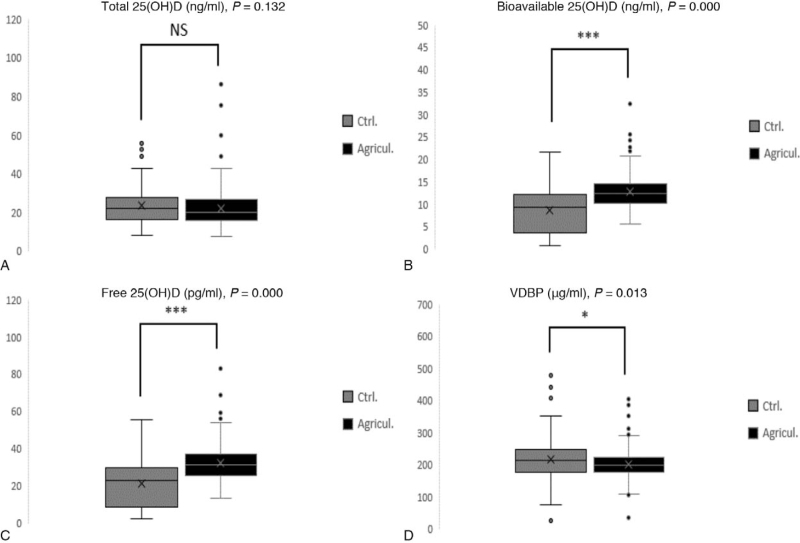
Comparisons of (A) total, (B) bioavailable, and (C) free 25(OH)D and (D) VDBP concentrations between control and agriculture groups. 25(OH)D = 25-hydroxy vitamin D, VDBP = vitamin D-binding protein.

The concentration of total 25(OH)D was not significantly different between groups (*P* = .132; Table 2 and Fig. 1). The concentrations of both bioavailable 25(OH)D (12.8 ± 3.7 vs 8.7 ± 5.1 ng/mL) and free 25(OH)D (32.4 ± 9.5 vs 21.2 ± 12.5 pg/mL) were significantly higher in the agriculture group than in the control group (both *P* = .000; Table 2 and Fig. 1). In contrast, the VDBP concentration was significantly lower in the agricultural group (201.8 ± 45.0 μg/mL) than in the control group (216.0 ± 68.2 μg/mL; *P* = .013; Table 2 and Fig. 1).

### 3.3 Association of menopause and agricultural work with osteopenia and osteoporosis

A log-linear model was used to determine the association between menopause and agricultural work and the occurrence of osteopenia and osteoporosis. Menopausal women had a 7.1-fold higher odds ratio of osteopenia than non-menopausal women (*P* = .000; Table 3). Although agricultural work was not associated with osteopenia (*P* = .684), it was associated with osteoporosis, resulting in a 4.3-fold increase in the odds ratio compared with non-agricultural work (*P* < .001; Table 3).

### 3.4 Univariable linear model for general characteristics and vitamin D status affecting bone health

The univariable linear model showed that all of the following factors were associated with femur neck BMD: age, BMI, smoking, agricultural work, menopause, bioavailable and free 25(OH)D, VDBP, calcium, and phosphate (Table 4). Interestingly, these same factors, excluding agricultural work, were also associated with the lumbar spine BMD (Table 4).

### 3.5 Multivariable linear model for vitamin D biomarkers affecting femur neck and lumbar spine (L1–L4) BMD

We used the coefficients of the multivariable linear model analysis to determine which vitamin D biomarkers affected bone health and evaluated how agricultural work affects bone health. Agricultural work, menopause, bioavailable 25(OH)D, and VDBP had an effect on femur neck bone health (*P* = .000, .006, .000, and .000, respectively), whereas menopause, bioavailable 25(OH)D, and VDBP affected lumbar spine bone health (*P* = .027, .009, and .000, respectively; Table 5).

## 4 Discussion

In this study, we investigated the bone health status of women working in agriculture compared with women in non-agricultural occupations to determine correlations between bone health and vitamin D biomarkers. Our data revealed no difference in the total 25(OH)D concentration between women working in agriculture and women in other occupations (Fig. 1). Interestingly, in agricultural workers, the concentrations of bioavailable 25(OH)D and free 25(OH)D were significantly higher, whereas VDBP was significantly lower than those measured in women working in other occupations (Fig. 1). In addition, the femur neck BMD was lower, and the probability of having osteoporosis increased among women working in agriculture compared with those in the control group (Table 3). Univariable and multivariable linear analyses showed that the vitamin D biomarkers that impacted BMD were bioavailable 25(OH)D and VDBP. To our knowledge, this study is the first study to investigate the association between occupation and female bone health and to analyze the association between bone health and specific vitamin D biomarkers.

Although no difference in lumbar BMD emerged between groups, the femur neck BMD differed significantly (Table 2), which was confirmed by univariable and multivariable linear model analysis (Table 4). As expected, the log-linear model analysis of the proportions of osteopenia in both groups showed that menopause increased the risk of osteopenia by 7.1-fold (Table 3). Interestingly, agricultural work did not increase the risk of osteopenia but did increase the risk of osteoporosis by 4.3-fold (Table 3). In general, agricultural work requires more physical activity than other occupations; however, Korean farmers often sit to work, and the sedentary work of these Korean farmers may influence femur BMD more than lumbar BMD. In support of this, a previous study linked bone development in childhood with lumbar BMD in adulthood, whereas only recent physical activity affected femur BMD.^[29]^ A lack of significant difference in lumbar BMD between occupational groups combined with the finding of a significantly deteriorated femur BMD may imply a differential influence on BMD mediated by occupation-associated physical activity. These effects may occur at different body parts and at different ages and may be associated with the processes of bone construction and bone aging.

Contrary to expectations, we found no significant difference in serum total 25(OH)D concentration between the agricultural and control groups (Fig. 1). Total 25(OH)D is converted from vitamin D by the liver after the skin is exposed to sunlight, and its concentration is closely related to the amount of sunlight exposure. The characteristic of Korean agriculture occurring mostly inside of greenhouses might explain the lack of difference in total 25(OH)D between occupations. Actual sunlight exposure did not differ significantly in agricultural workers compared with occupational groups working in other indoor areas due to the use of sunscreen or sunscreen clothing during work. In contrast, fishermen had a higher serum total 25(OH)D compared with individuals in other occupations.^[30]^ All subjects in this study resided in the same area and participated for the same time period, which minimized differences in dietary habits, latitude, and seasonal effects between groups.

Total 25(OH)D did not differ between groups, but bioavailable 25(OH)D was significantly different between the 2 groups. Bioavailable 25(OH)D is defined as the level of vitamin D free from the transporter, VDBP, and is thought to be more physiologically and clinically important than total 25(OH)D levels.^[18,31,32]^ Thus, we hypothesize that higher levels of VDBP would result in less biologically available vitamin D in circulation. Conversely, lower VDBP levels would result in more bioavailable 25(OH)D. In our study, agricultural workers had higher bioavailable 25(OH)D and lower VDBP concentrations than control workers. The higher bioavailable 25(OH)D concentration in the agriculture group could be attributed to the lower VDBP concentration.

Previous studies showed a positive correlation between vitamin D concentration and bone health, but in our study, agricultural workers showed a higher bioavailable 25(OH)D concentration, a lower VDBP concentration, and decreased bone health^[28,33,34]^ (Fig. 1, Table 2). VDBP has been reported as a potential biomarker associated with low BMD in a previous serum proteomic study.^[35]^ Consistent with this finding, agricultural workers in our study had a lower BMD and higher risk of osteopenia and osteoporosis compared with the control group. Taken together, these results suggest that VDBP may play an important role in bone metabolism. Furthermore, we found that the incidence of lumbar osteoporosis increased in women working in agriculture after menopause.

Several limitations to this study may affect the results. First, environmental factors that can influence vitamin D concentrations were not surveyed, such as diet, time spent outdoors, the use of sunscreen, and vitamin D supplement intake. Second, the serum levels of vitamin D and VDBP were only measured at 1 time point, with no follow-up. Last, the VDBP genotype of participants was not analyzed, which could explain some differences in the VDBP levels. Three major polymorphic forms of VDBP have been identified, GC1F, GC1S, and GC2 (rs7041 and rs4588)^[36]^; however, these were not considered in the present study.

In conclusion, serum bioavailable 25(OH)D concentrations were higher, and VDBP levels were lower in women working in agricultural occupations than in women working in control occupations. These differences affected the femur neck BMD, as assessed by univariable and multivariable linear model analysis. No changes in lumbar BMD were found between groups, but femur BMD was lower in agricultural workers than in control workers. Women working in agriculture have a higher probability of having osteoporosis compared with the control group. VDBP may play a role in the reduced femur neck BMD in the agricultural group, and VDBP is an important variable that determines bioavailable 25(OH)D levels, which are currently derived using equations and not measured directly. We propose the measurement of VDBP levels as a simple, non-invasive alternative approach to dual-energy X-ray absorptiometry for the measurement of bone health status, which will decrease both radiation exposure and costs.

## Author contributions

**Conceptualization:** Min-Chul Cho, Won Jun Choi.

**Data curation:** Jeong Kyu Shin, Soon Ae Lee, In Ae Cho, Hyen Chul Jo.

**Formal analysis:** Min-Chul Cho, Seung Chan Kim, Won Jun Choi.

**Funding acquisition:** Min-Chul Cho.

**Investigation:** Ki Soo Park.

**Methodology:** Min-Chul Cho, Won Jun Choi.

**Resources:** Ki Soo Park.

**Software:** Seung Chan Kim.

**Validation:** Ki Soo Park.

**Visualization:** Seung Chan Kim.

**Writing – original draft:** Min-Chul Cho.

**Writing – review & editing:** Won Jun Choi.
